# 3-Isopropyl-2,6-bis­(4-meth­oxy­phen­yl)­piperidin-4-one

**DOI:** 10.1107/S1600536812030966

**Published:** 2012-07-14

**Authors:** K. Ravichandran, S. Sethuvasan, K. Thirunavukarasu, S. Ponnuswamy, M. N. Ponnuswamy

**Affiliations:** a Centre of Advanced Study in Crystallography and Biophysics, University of Madras, Guindy Campus, Chennai 600 025, India; b Department of Chemistry, Government Arts College (Autonomous), Coimbatore 641 018, India

## Abstract

In the title compound, C_22_H_27_NO_3_, the piperidine ring adopts a slightly distorted chair conformation. The dihedral angle between the two aromatic rings is 60.4 (1)°. In the crystal, the amino group forms a rather long N—H⋯O contact to a methoxy O atom. There are also C—H⋯O interactions present.

## Related literature

For the biological activity of piperidine derivatives, see: Bochringer & Soehne (1961 ▶); El-Subbagh *et al.* (2000 ▶); Ganellin & Spickett (1965 ▶); Hagenbach & Gysin (1952 ▶); Jerom & Spencer (1988 ▶); Katritzky & Fan (1990 ▶); Perumal *et al.* (2001 ▶); Ravindran *et al.* (1991 ▶); Severs *et al.* (1965 ▶). For puckering parameters, see: Cremer & Pople (1975 ▶). For asymmetry parameters, see: Nardelli (1983 ▶). For hydrogen-bond motifs, see: Bernstein *et al.* (1995 ▶).
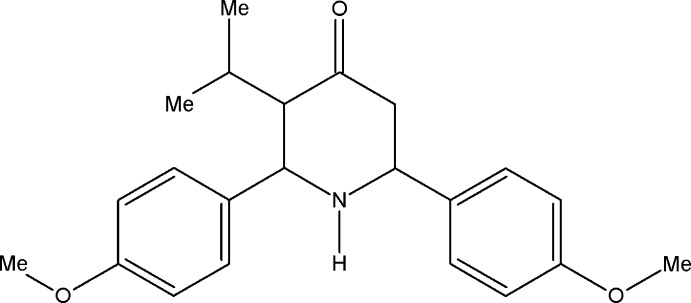


## Experimental

### 

#### Crystal data

C_22_H_27_NO_3_
*M* *_r_* = 353.45
Orthorhombic, 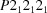

*a* = 7.5547 (3) Å
*b* = 11.8792 (6) Å
*c* = 22.1103 (10) Å
*V* = 1984.26 (16) Å^3^
*Z* = 4
Mo *K*α radiation
μ = 0.08 mm^−1^
*T* = 293 K
0.22 × 0.20 × 0.18 mm

#### Data collection

Bruker SMART APEX CCD detector diffractometer
Absorption correction: multi-scan (*SADABS*; Bruker, 2008 ▶) *T*_min_ = 0.983, *T*_max_ = 0.986
10956 measured reflections
2801 independent reflections
1835 reflections with *I* > 2σ(*I*)
*R*_int_ = 0.038

#### Refinement

*R*[*F* ^2^ > 2σ(*F* ^2^)] = 0.044
*wR*(*F* ^2^) = 0.121
*S* = 1.03
2801 reflections
241 parameters
H atoms treated by a mixture of independent and constrained refinement
Δρ_max_ = 0.17 e Å^−3^
Δρ_min_ = −0.13 e Å^−3^

### 

Data collection: *APEX2* (Bruker, 2008 ▶); cell refinement: *SAINT* (Bruker, 2008 ▶); data reduction: *SAINT*; program(s) used to solve structure: *SHELXS97* (Sheldrick, 2008 ▶); program(s) used to refine structure: *SHELXL97* (Sheldrick, 2008 ▶); molecular graphics: *ORTEP-3* (Farrugia, 1997 ▶); software used to prepare material for publication: *SHELXL97* and *PLATON* (Spek, 2009 ▶).

## Supplementary Material

Crystal structure: contains datablock(s) global, I. DOI: 10.1107/S1600536812030966/bt5928sup1.cif

Structure factors: contains datablock(s) I. DOI: 10.1107/S1600536812030966/bt5928Isup2.hkl

Supplementary material file. DOI: 10.1107/S1600536812030966/bt5928Isup3.cml

Additional supplementary materials:  crystallographic information; 3D view; checkCIF report

## Figures and Tables

**Table 1:** Hydrogen-bond geometry (Å, °)

*D*—H⋯*A*	*D*—H	H⋯*A*	*D*⋯*A*	*D*—H⋯*A*
N1—H1⋯O1^i^	0.90 (2)	2.66 (2)	3.538 (2)	167.4 (17)
C16—H16*A*⋯O1^ii^	0.96	2.57	3.474 (4)	156

Symmetry codes: (i) 
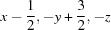
; (ii) 
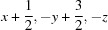
.

**Fig. 1.**
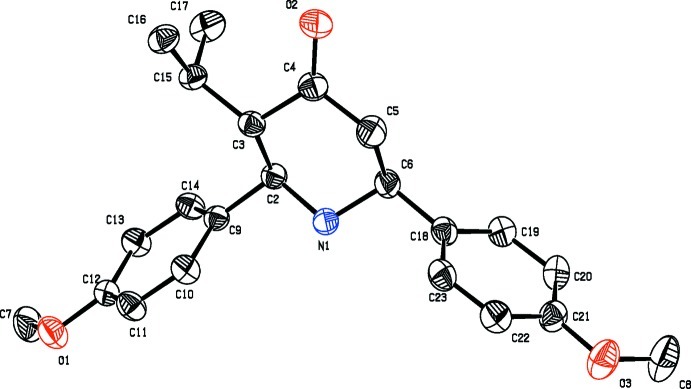
The molecular structure of the title compound, showing the atomic numbering and displacement ellipsoids drawn at 30% probability level.

**Fig. 2.**
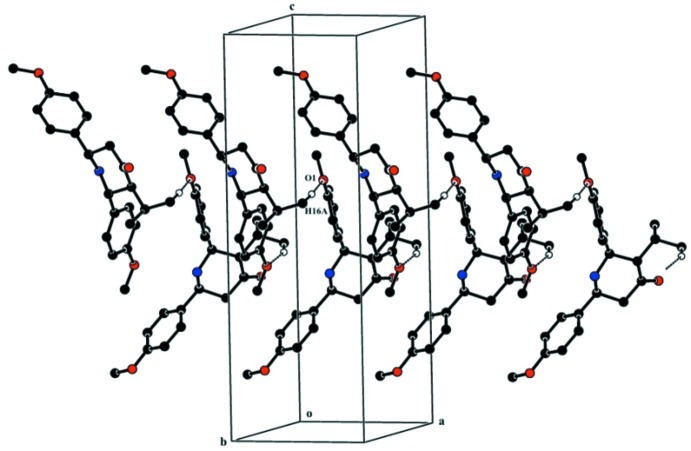
The crystal packing of the molecules viewed down b axis. H atoms not involved in hydrogen bonding (dashed lines) have been omitted for clarity.

## supplementary crystallographic information

### Comment

In the family of heterocyclic compounds, piperidin-4-ones possess varied biological properties such as antiviral, antitumour (El-Subbagh *et al.*, 2000), analgesic (Jerom & Spencer, 1988), local anaesthetic (Perumal *et al.*, 2001; Hagenbach & Gysin, 1952), anti-inflammatory and anticancer activities (Katritzky & Fan, 1990). Several 2,6-disubstituted piperidines are found to be useful as tranquillisers (Bochringer & Soehne, 1961) and possess hypotensive activity (Severs *et al.*, 1965), a combination of stimulant and depressant effects on the central nervous system (Ganellin & Spickett, 1965). Also the substitution of methoxy phenyl groups at 2,6-positions is found to be active against *CNS* subpanels. In addition, the bulkiness of the subtituent in different positons of the piperidine ring leads to the decrease in carcinogenecity (Ravindran *et al.*, 1991). In view of the importance, the crystallographic study of the title compound has been carried out to establish the molecular structure and conformation.

The *ORTEP* plot of the molecule is shown in Fig. 1. The piperidine ring adopts distorted chair conformation. The puckering (Cremer & Pople, 1975) and the asymmetry parameters (Nardelli, 1983) are: q_2_=0.121 (3) Å, q_3_ = 0.569 (3) Å, φ_2_ = 7.6 (1)° and Δ_s_(N1 & C4) = 1.3 (2)°. The sum of the bond angles around the atom N1 [333.5°] is in accordance with *sp^3^* hybridization.

The best plane of the piperidine ring is oriented with respect to the phenyl rings (C9—C14) & (C18—C23) at angles of 82.8 (1)° & 86.2 (1)°, respectively. The two phenyl rings are set apart with an angle of 60.4 (1)°. The methoxy groups substituted at the phenyl rings are coplanar, which can be seen from the torsion angles of [C13—C12—O1—C7=] 4.4 (4)° for (C9—C14) ring and [C20—C21—O3—C8 =] 1.6 (5)° for (C18—C23) ring.

The packing of the molecules is stabilized by a rather long N-H···O contact and by C—H···O interactions in addition to van der Waals forces.

### Experimental

Ammonium acetate (100 mmol), anisaldehyde (200 mmol) and isobutylmethylketone (100 mmol) in ethanol (30 ml) were heated on a hot plate at 50–55° C and after the completion of reaction, water was added and extracted with ether, dried and recrystallized from ethanol.

### Refinement

Due to the absence of anomalous scatterers, the absolute configuration could not be determined and Friedel pairs were merged. C-bound H atoms were positioned geometrically (C–H = 0.93–0.97 Å) and allowed to ride on their parent atoms, with *U*_iso_(H) = 1.5*U*_eq_(C) for methyl H atoms and 1.2*U*_eq_(C) for all other H atoms. The H atom bonded to N was freely refined.

### Crystal data

**Table:**

C_22_H_27_NO_3_	*F*(000) = 760
*M**_r_* = 353.45	*D*_x_ = 1.183 Mg m^−^^3^
Orthorhombic, *P*2_1_2_1_2_1_	Mo *K*α radiation, λ = 0.71073 Å
Hall symbol: P 2ac 2ab	Cell parameters from 2938 reflections
*a* = 7.5547 (3) Å	θ = 1.8–28.3°
*b* = 11.8792 (6) Å	µ = 0.08 mm^−^^1^
*c* = 22.1103 (10) Å	*T* = 293 K
*V* = 1984.26 (16) Å^3^	Black, white crystalline
*Z* = 4	0.22 × 0.20 × 0.18 mm

### Data collection

**Table:**

Bruker SMART APEX CCD detector diffractometer	2801 independent reflections
Radiation source: fine-focus sealed tube	1835 reflections with *I* > 2σ(*I*)
Graphite monochromator	*R*_int_ = 0.038
ω scans	θ_max_ = 28.3°, θ_min_ = 1.8°
Absorption correction: multi-scan (*SADABS*; Bruker, 2008)	*h* = −10→9
*T*_min_ = 0.983, *T*_max_ = 0.986	*k* = −15→8
10956 measured reflections	*l* = −27→29

### Refinement

**Table:**

Refinement on *F*^2^	Primary atom site location: structure-invariant direct methods
Least-squares matrix: full	Secondary atom site location: difference Fourier map
*R*[*F*^2^ > 2σ(*F*^2^)] = 0.044	Hydrogen site location: inferred from neighbouring sites
*wR*(*F*^2^) = 0.121	H atoms treated by a mixture of independent and constrained refinement
*S* = 1.03	*w* = 1/[σ^2^(*F*_o_^2^) + (0.0621*P*)^2^ + 0.0599*P*] where *P* = (*F*_o_^2^ + 2*F*_c_^2^)/3
2801 reflections	(Δ/σ)_max_ = 0.015
241 parameters	Δρ_max_ = 0.17 e Å^−^^3^
0 restraints	Δρ_min_ = −0.13 e Å^−^^3^

### Special details

**Table:**

Geometry. All e.s.d.'s (except the e.s.d. in the dihedral angle between two l.s. planes) are estimated using the full covariance matrix. The cell e.s.d.'s are taken into account individually in the estimation of e.s.d.'s in distances, angles and torsion angles; correlations between e.s.d.'s in cell parameters are only used when they are defined by crystal symmetry. An approximate (isotropic) treatment of cell e.s.d.'s is used for estimating e.s.d.'s involving l.s. planes.
Refinement. Refinement of *F*^2^ against ALL reflections. The weighted *R*-factor *wR* and goodness of fit *S* are based on *F*^2^, conventional *R*-factors *R* are based on *F*, with *F* set to zero for negative *F*^2^. The threshold expression of *F*^2^ > σ(*F*^2^) is used only for calculating *R*-factors(gt) *etc*. and is not relevant to the choice of reflections for refinement. *R*-factors based on *F*^2^ are statistically about twice as large as those based on *F*, and *R*- factors based on ALL data will be even larger.

### Fractional atomic coordinates and isotropic or equivalent isotropic displacement parameters (Å^2^)

**Table:**

	*x*	*y*	*z*	*U*_iso_*/*U*_eq_	
H1	0.161 (4)	0.568 (2)	0.1225 (11)	0.049 (7)*	
O1	0.3703 (3)	0.80940 (17)	−0.10981 (8)	0.0655 (6)	
O2	0.6143 (3)	0.2871 (2)	0.15552 (10)	0.0773 (7)	
O3	−0.2117 (3)	0.5919 (2)	0.37078 (10)	0.0865 (8)	
N1	0.2620 (3)	0.53392 (18)	0.13369 (9)	0.0446 (5)	
C2	0.3533 (3)	0.4891 (2)	0.08036 (10)	0.0433 (6)	
H2	0.2910	0.4212	0.0669	0.052*	
C3	0.5444 (3)	0.4554 (2)	0.09959 (11)	0.0451 (6)	
H3	0.5993	0.5241	0.1155	0.054*	
C4	0.5323 (4)	0.3749 (3)	0.15235 (13)	0.0556 (7)	
C5	0.4118 (4)	0.4096 (3)	0.20247 (12)	0.0676 (9)	
H5A	0.4656	0.4711	0.2247	0.081*	
H5B	0.3966	0.3470	0.2302	0.081*	
C6	0.2300 (3)	0.4469 (2)	0.17908 (12)	0.0507 (6)	
H6	0.1737	0.3825	0.1592	0.061*	
C7	0.3092 (5)	0.7822 (3)	−0.16890 (12)	0.0731 (9)	
H7A	0.3785	0.7215	−0.1850	0.110*	
H7B	0.3206	0.8469	−0.1946	0.110*	
H7C	0.1872	0.7599	−0.1670	0.110*	
C8	−0.3714 (6)	0.5342 (4)	0.38404 (18)	0.1054 (14)	
H8A	−0.4477	0.5363	0.3493	0.158*	
H8B	−0.4292	0.5699	0.4176	0.158*	
H8C	−0.3453	0.4574	0.3941	0.158*	
C9	0.3520 (3)	0.5733 (2)	0.02940 (10)	0.0413 (5)	
C10	0.4059 (3)	0.6843 (2)	0.03831 (12)	0.0508 (6)	
H10	0.4390	0.7076	0.0768	0.061*	
C11	0.4112 (4)	0.7599 (2)	−0.00879 (13)	0.0555 (7)	
H11	0.4486	0.8333	−0.0019	0.067*	
C12	0.3614 (3)	0.7275 (2)	−0.06612 (11)	0.0470 (6)	
C13	0.3080 (4)	0.6181 (2)	−0.07623 (11)	0.0518 (7)	
H13	0.2749	0.5950	−0.1148	0.062*	
C14	0.3041 (3)	0.5433 (2)	−0.02846 (11)	0.0493 (6)	
H14	0.2676	0.4697	−0.0357	0.059*	
C15	0.6625 (3)	0.4165 (2)	0.04695 (12)	0.0470 (6)	
H15	0.6556	0.4757	0.0162	0.056*	
C16	0.8571 (3)	0.4099 (3)	0.06599 (13)	0.0578 (7)	
H16A	0.8910	0.4792	0.0851	0.087*	
H16B	0.9296	0.3976	0.0309	0.087*	
H16C	0.8728	0.3488	0.0939	0.087*	
C17	0.6047 (4)	0.3087 (3)	0.01612 (14)	0.0691 (8)	
H17A	0.4828	0.3150	0.0043	0.104*	
H17B	0.6181	0.2467	0.0436	0.104*	
H17C	0.6765	0.2961	−0.0191	0.104*	
C18	0.1107 (3)	0.4866 (2)	0.22900 (11)	0.0489 (6)	
C19	−0.0469 (3)	0.4320 (2)	0.24044 (12)	0.0521 (6)	
H19	−0.0796	0.3720	0.2159	0.063*	
C20	−0.1579 (4)	0.4634 (3)	0.28710 (12)	0.0577 (7)	
H20	−0.2627	0.4243	0.2939	0.069*	
C21	−0.1124 (4)	0.5531 (3)	0.32367 (12)	0.0579 (7)	
C22	0.0451 (4)	0.6089 (3)	0.31305 (13)	0.0625 (8)	
H22	0.0774	0.6689	0.3377	0.075*	
C23	0.1547 (4)	0.5767 (2)	0.26643 (13)	0.0577 (7)	
H23	0.2597	0.6156	0.2598	0.069*	

### Atomic displacement parameters (Å^2^)

**Table:**

	*U*^11^	*U*^22^	*U*^33^	*U*^12^	*U*^13^	*U*^23^
O1	0.0953 (15)	0.0511 (11)	0.0502 (11)	−0.0043 (11)	0.0027 (10)	0.0050 (9)
O2	0.0696 (13)	0.0697 (14)	0.0927 (15)	0.0253 (12)	0.0052 (11)	0.0272 (12)
O3	0.0879 (16)	0.1049 (19)	0.0665 (14)	−0.0023 (15)	0.0205 (12)	−0.0176 (13)
N1	0.0394 (10)	0.0469 (13)	0.0475 (12)	0.0030 (10)	−0.0020 (9)	0.0051 (10)
C2	0.0364 (10)	0.0433 (14)	0.0503 (13)	−0.0006 (10)	−0.0025 (10)	0.0002 (11)
C3	0.0401 (11)	0.0420 (14)	0.0532 (14)	0.0022 (10)	−0.0056 (11)	0.0021 (13)
C4	0.0427 (13)	0.0603 (18)	0.0638 (17)	0.0085 (13)	−0.0080 (12)	0.0113 (15)
C5	0.0638 (17)	0.085 (2)	0.0543 (16)	0.0188 (17)	−0.0014 (14)	0.0201 (16)
C6	0.0504 (13)	0.0529 (16)	0.0487 (14)	0.0018 (12)	0.0016 (11)	0.0082 (13)
C7	0.108 (2)	0.068 (2)	0.0435 (16)	0.0003 (19)	0.0086 (17)	0.0070 (15)
C8	0.096 (3)	0.137 (4)	0.083 (2)	−0.012 (3)	0.038 (2)	−0.014 (2)
C9	0.0333 (10)	0.0407 (14)	0.0499 (14)	0.0004 (10)	−0.0019 (10)	−0.0008 (11)
C10	0.0588 (15)	0.0479 (16)	0.0457 (14)	−0.0043 (13)	−0.0080 (12)	−0.0018 (13)
C11	0.0693 (17)	0.0400 (15)	0.0572 (16)	−0.0103 (13)	−0.0047 (14)	−0.0029 (13)
C12	0.0519 (13)	0.0414 (16)	0.0478 (14)	0.0024 (12)	0.0057 (11)	0.0012 (12)
C13	0.0661 (16)	0.0472 (16)	0.0420 (14)	0.0008 (12)	−0.0071 (12)	−0.0036 (12)
C14	0.0567 (14)	0.0383 (14)	0.0529 (15)	−0.0021 (11)	−0.0056 (12)	−0.0028 (12)
C15	0.0413 (11)	0.0454 (14)	0.0543 (14)	0.0048 (11)	−0.0024 (11)	−0.0031 (12)
C16	0.0431 (13)	0.0596 (18)	0.0706 (18)	0.0027 (12)	−0.0004 (13)	−0.0089 (15)
C17	0.0547 (16)	0.0677 (19)	0.085 (2)	0.0008 (15)	−0.0062 (15)	−0.0205 (17)
C18	0.0478 (13)	0.0529 (16)	0.0461 (13)	0.0025 (12)	−0.0039 (11)	0.0104 (13)
C19	0.0509 (13)	0.0556 (16)	0.0498 (14)	−0.0027 (13)	−0.0045 (12)	0.0008 (13)
C20	0.0513 (13)	0.073 (2)	0.0485 (14)	−0.0068 (14)	0.0014 (12)	0.0042 (14)
C21	0.0625 (15)	0.0669 (19)	0.0443 (14)	0.0046 (14)	0.0016 (13)	0.0002 (14)
C22	0.0728 (18)	0.0581 (19)	0.0566 (17)	−0.0064 (15)	−0.0047 (15)	−0.0039 (15)
C23	0.0561 (15)	0.0556 (17)	0.0615 (17)	−0.0095 (14)	−0.0029 (13)	0.0068 (14)

### Geometric parameters (Å, º)

**Table:**

O1—C12	1.373 (3)	C10—C11	1.376 (4)
O1—C7	1.423 (3)	C10—H10	0.9300
O2—C4	1.215 (3)	C11—C12	1.377 (4)
O3—C21	1.364 (3)	C11—H11	0.9300
O3—C8	1.419 (5)	C12—C13	1.379 (4)
N1—C6	1.461 (3)	C13—C14	1.381 (4)
N1—C2	1.466 (3)	C13—H13	0.9300
N1—H1	0.90 (3)	C14—H14	0.9300
C2—C9	1.507 (3)	C15—C17	1.515 (4)
C2—C3	1.557 (3)	C15—C16	1.531 (3)
C2—H2	0.9800	C15—H15	0.9800
C3—C4	1.512 (4)	C16—H16A	0.9600
C3—C15	1.538 (3)	C16—H16B	0.9600
C3—H3	0.9800	C16—H16C	0.9600
C4—C5	1.493 (4)	C17—H17A	0.9600
C5—C6	1.533 (4)	C17—H17B	0.9600
C5—H5A	0.9700	C17—H17C	0.9600
C5—H5B	0.9700	C18—C19	1.380 (4)
C6—C18	1.501 (4)	C18—C23	1.392 (4)
C6—H6	0.9800	C19—C20	1.381 (4)
C7—H7A	0.9600	C19—H19	0.9300
C7—H7B	0.9600	C20—C21	1.382 (4)
C7—H7C	0.9600	C20—H20	0.9300
C8—H8A	0.9600	C21—C22	1.382 (4)
C8—H8B	0.9600	C22—C23	1.377 (4)
C8—H8C	0.9600	C22—H22	0.9300
C9—C14	1.377 (3)	C23—H23	0.9300
C9—C10	1.393 (4)		
			
C12—O1—C7	118.0 (2)	C9—C10—H10	119.4
C21—O3—C8	117.5 (3)	C10—C11—C12	120.4 (3)
C6—N1—C2	111.9 (2)	C10—C11—H11	119.8
C6—N1—H1	111.7 (16)	C12—C11—H11	119.8
C2—N1—H1	109.9 (16)	O1—C12—C11	115.9 (2)
N1—C2—C9	110.95 (19)	O1—C12—C13	124.6 (2)
N1—C2—C3	108.06 (19)	C11—C12—C13	119.5 (2)
C9—C2—C3	112.40 (18)	C12—C13—C14	119.3 (2)
N1—C2—H2	108.4	C12—C13—H13	120.4
C9—C2—H2	108.4	C14—C13—H13	120.4
C3—C2—H2	108.4	C9—C14—C13	122.6 (2)
C4—C3—C15	115.4 (2)	C9—C14—H14	118.7
C4—C3—C2	108.5 (2)	C13—C14—H14	118.7
C15—C3—C2	114.1 (2)	C17—C15—C16	111.0 (2)
C4—C3—H3	106.0	C17—C15—C3	115.3 (2)
C15—C3—H3	106.0	C16—C15—C3	111.4 (2)
C2—C3—H3	106.0	C17—C15—H15	106.2
O2—C4—C5	120.4 (3)	C16—C15—H15	106.2
O2—C4—C3	123.9 (3)	C3—C15—H15	106.2
C5—C4—C3	115.8 (2)	C15—C16—H16A	109.5
C4—C5—C6	112.1 (2)	C15—C16—H16B	109.5
C4—C5—H5A	109.2	H16A—C16—H16B	109.5
C6—C5—H5A	109.2	C15—C16—H16C	109.5
C4—C5—H5B	109.2	H16A—C16—H16C	109.5
C6—C5—H5B	109.2	H16B—C16—H16C	109.5
H5A—C5—H5B	107.9	C15—C17—H17A	109.5
N1—C6—C18	112.5 (2)	C15—C17—H17B	109.5
N1—C6—C5	106.8 (2)	H17A—C17—H17B	109.5
C18—C6—C5	112.4 (2)	C15—C17—H17C	109.5
N1—C6—H6	108.4	H17A—C17—H17C	109.5
C18—C6—H6	108.4	H17B—C17—H17C	109.5
C5—C6—H6	108.4	C19—C18—C23	117.3 (2)
O1—C7—H7A	109.5	C19—C18—C6	120.4 (2)
O1—C7—H7B	109.5	C23—C18—C6	122.4 (2)
H7A—C7—H7B	109.5	C18—C19—C20	122.3 (3)
O1—C7—H7C	109.5	C18—C19—H19	118.9
H7A—C7—H7C	109.5	C20—C19—H19	118.9
H7B—C7—H7C	109.5	C19—C20—C21	119.6 (3)
O3—C8—H8A	109.5	C19—C20—H20	120.2
O3—C8—H8B	109.5	C21—C20—H20	120.2
H8A—C8—H8B	109.5	O3—C21—C22	116.2 (3)
O3—C8—H8C	109.5	O3—C21—C20	124.8 (3)
H8A—C8—H8C	109.5	C22—C21—C20	119.0 (3)
H8B—C8—H8C	109.5	C23—C22—C21	120.8 (3)
C14—C9—C10	117.0 (2)	C23—C22—H22	119.6
C14—C9—C2	121.6 (2)	C21—C22—H22	119.6
C10—C9—C2	121.3 (2)	C22—C23—C18	121.0 (3)
C11—C10—C9	121.3 (2)	C22—C23—H23	119.5
C11—C10—H10	119.4	C18—C23—H23	119.5
			
C6—N1—C2—C9	168.49 (19)	C10—C11—C12—C13	−0.7 (4)
C6—N1—C2—C3	−67.9 (2)	O1—C12—C13—C14	−179.8 (2)
N1—C2—C3—C4	54.9 (3)	C11—C12—C13—C14	0.5 (4)
C9—C2—C3—C4	177.7 (2)	C10—C9—C14—C13	−0.1 (4)
N1—C2—C3—C15	−174.9 (2)	C2—C9—C14—C13	−177.6 (2)
C9—C2—C3—C15	−52.1 (3)	C12—C13—C14—C9	−0.1 (4)
C15—C3—C4—O2	3.0 (4)	C4—C3—C15—C17	61.6 (3)
C2—C3—C4—O2	132.5 (3)	C2—C3—C15—C17	−65.1 (3)
C15—C3—C4—C5	−177.4 (2)	C4—C3—C15—C16	−66.0 (3)
C2—C3—C4—C5	−47.9 (3)	C2—C3—C15—C16	167.3 (2)
O2—C4—C5—C6	−131.9 (3)	N1—C6—C18—C19	120.8 (3)
C3—C4—C5—C6	48.5 (4)	C5—C6—C18—C19	−118.7 (3)
C2—N1—C6—C18	−170.4 (2)	N1—C6—C18—C23	−61.0 (3)
C2—N1—C6—C5	65.9 (3)	C5—C6—C18—C23	59.5 (3)
C4—C5—C6—N1	−53.8 (3)	C23—C18—C19—C20	−0.5 (4)
C4—C5—C6—C18	−177.6 (3)	C6—C18—C19—C20	177.9 (2)
N1—C2—C9—C14	−131.8 (2)	C18—C19—C20—C21	0.7 (4)
C3—C2—C9—C14	107.1 (3)	C8—O3—C21—C22	−178.0 (3)
N1—C2—C9—C10	50.7 (3)	C8—O3—C21—C20	1.6 (5)
C3—C2—C9—C10	−70.4 (3)	C19—C20—C21—O3	179.7 (3)
C14—C9—C10—C11	−0.2 (4)	C19—C20—C21—C22	−0.8 (4)
C2—C9—C10—C11	177.4 (2)	O3—C21—C22—C23	−179.7 (3)
C9—C10—C11—C12	0.6 (4)	C20—C21—C22—C23	0.7 (4)
C7—O1—C12—C11	−175.9 (3)	C21—C22—C23—C18	−0.5 (4)
C7—O1—C12—C13	4.4 (4)	C19—C18—C23—C22	0.4 (4)
C10—C11—C12—O1	179.6 (2)	C6—C18—C23—C22	−177.9 (3)

### Hydrogen-bond geometry (Å, º)

**Table:**

*D*—H···*A*	*D*—H	H···*A*	*D*···*A*	*D*—H···*A*
N1—H1···O1^i^	0.90 (2)	2.66 (2)	3.538 (2)	167.4 (17)
C16—H16*A*···O1^ii^	0.96	2.57	3.474 (4)	156

Symmetry codes: (i) *x*−1/2, −*y*+3/2, −*z*; (ii) *x*+1/2, −*y*+3/2, −*z*.
